# Potential cognitive and neural benefits of a computerised cognitive training programme based on Structure Learning in healthy adults: study protocol for a randomised controlled trial

**DOI:** 10.1186/s13063-023-07551-2

**Published:** 2023-08-11

**Authors:** Chia-Lun Liu, Xiaoqin Cheng, Boon Linn Choo, Min Hong, Jia Li Teo, Wei Ler Koo, Jia Yuan Janet Tan, Marisha Barth Ubrani, John Suckling, Balázs Gulyás, Victoria Leong, Zoe Kourtzi, Barbara Sahakian, Trevor Robbins, Annabel Shen-Hsing Chen

**Affiliations:** 1 Centre for Research and Development in Learning (CRADLE), Nanyang Technological University, Singapore, Singapore; 2 Department of Psychology, University of Innsbruck, Innsbruck, Austria; 3 School of Social Sciences, Nanyang Technological University, Singapore, Singapore; 4 Department of Psychiatry, University of Cambridge, Cambridge, CB2 0SZ UK; 5 Lee Kong Chian School of Medicine, Nanyang Technological University, Singapore, Singapore; 6 Department of Psychology, University of Cambridge, Cambridge, CB2 3EB UK; 7 Behavioural and Clinical Neuroscience Institute, University of Cambridge, Cambridge, CB2 3EB UK; 8 National Institute of Education, Nanyang Technological University, Singapore, Singapore

**Keywords:** Structure learning, Cognitive flexibility, Magnetic resonance imaging, Intervention, Statistical learning, Neuropsychological model of cognitive flexibility, Exploration, Exploitation

## Abstract

**Background:**

Cognitive flexibility refers to the capacity to shift between conceptual representations particularly in response to changes in instruction and feedback. It enables individuals to swiftly adapt to changes in their environment and has significant implications for learning. The present study focuses on investigating changes in cognitive flexibility following an intervention programme—Structure Learning training.

**Methods:**

Participants are pseudo-randomised to either the Training or Control group, while matched on age, sex, intelligence and cognitive flexibility performance. In the Training group, participants undergo around 2 weeks of training (at least 13 sessions) on Structure Learning. In the Control group, participants do not have to undergo any training and are never exposed to the Structure Learning task. The effects of Structure Learning training are investigated at both the behavioural and neural level. We measured covariates that can influence an individual’s training performance before the training phase and outcome measures that can potentially show training benefits after the training phase. At the behavioural level, we investigated outcomes in both cognitive and social aspects with a primary focus on executive functions. At the neural level, we employed a multimodality approach and investigated potential changes to functional connectivity patterns, neurometabolite concentration in the frontal brain regions, and brain microstructure and myelination.

**Discussion:**

We reported the development of a novel training programme based on Structure Learning that aims to hone a general learning ability to potentially achieve extensive transfer benefits across various cognitive constructs. Potential transfer benefits can be exhibited through better performance in outcome measures between Training and Control participants, and positive associations between training performance and outcomes after the training in Training participants. Moreover, we attempt to substantiate behavioural findings with evidence of neural changes across different imaging modalities by the Structure Learning training.

**Trial registration:**

National Institutes of Health U.S. National Library of Medicine ClinicalTrials.gov NCT05611788. Registered on 7 November 2022. Protocol version: 11 May 2023.

**Supplementary Information:**

The online version contains supplementary material available at 10.1186/s13063-023-07551-2.

## Administrative information

Note: the numbers in curly brackets in this protocol refer to SPIRIT checklist item numbers. The order of the items has been modified to group similar items (see http://www.equator-network.org/reporting-guidelines/spirit-2013-statement-defining-standard-protocol-items-for-clinical-trials/).

**Table:** 

Title {1}	Potential cognitive and neural benefits of a computerised cognitive training programme based on Structure Learning in healthy adults: Study protocol for a randomised controlled trial
Trial registration {2a and 2b}.	The current intervention programme (Structure Learning Training and Cognitive Flexibility) has been pre-registered on the National Institutes of Health U.S. National Library of Medicine ClinicalTrials.gov NCT05611788. Registered on 7 November 2022. https://clinicaltrials.gov/ct2/show/NCT05611788.
Protocol version {3}	Protocol Version 1 (2022-November-7): First public release of protocol on ClinicalTrials.gov after resolution of MRI technical issues. Protocol Version 2 (2023-June-2): First submission to Trials and includes detailed outline of data analysis and statistical plan
Funding {4}	This research is supported by the National Research Foundation, Prime Minister’s Office, Singapore under its Campus for Research Excellence and Technological Enterprise Science of Learning (NRF-CREATE SoL) Programme with the funding administered by the Cambridge Centre for Advanced Research and Education in Singapore Ltd. (CARES) and housed at the Centre for Research and Development in Learning. (CRADLE@NTU).
Author details {5a}	Chia-Lun Liu^1^, Xiaoqin Cheng^1,8^, Boon Linn Choo^1^, Min Hong^1^, Jia Li Teo^1,2^, Wei Ler Koo^1^, Jia Yuan Janet Tan^1^, Marisha Barth Ubrani^1^, John Suckling^5^, Balázs Gulyás^3^, Victoria Leong^1,2,3^, Zoe Kourtzi^6^, Barbara Sahakian^5^, Trevor Robbins^6,7^, Annabel Shen-Hsing Chen^1,2,3,4^. 1. Centre for Research and Development in Learning (CRADLE), Nanyang Technological University, Singapore, Singapore. 2. School of Social Sciences, Nanyang Technological University, Singapore, Singapore. 3. Lee Kong Chian School of Medicine, Nanyang Technological University, Singapore, Singapore. 4. National Institute of Education, Nanyang Technological University, Singapore, Singapore. 5. Department of Psychiatry, University of Cambridge, Cambridge, CB2 0SZ, United Kingdom. 6. Department of Psychology, University of Cambridge, Cambridge, CB2 3EB, United Kingdom. 7. Behavioural and Clinical Neuroscience Institute, University of Cambridge, Cambridge CB2 3EB, UK. 8. Department of Psychology, University of Innsbruck, Innsbruck, Austria.
Name and contact information for the trial sponsor {5b}	Trial sponsor: National Research Foundation, Prime Minister’s Office, Singapore. Grant administrator: Cambridge Centre for Advanced Research and Education in Singapore Ltd. (CARES) Address: Cambridge CARES Office, 1 CREATE Way #05–05 CREATE Tower, Singapore 138,602 Contact: + 65 6601 5445 Email: enquiries@cares.cam.ac.uk
Role of sponsor {5c}	The study funders did not and will not have any role in the study design, collection, management, analysis and interpretation of data, writing of the report and the decision to submit the report for publication.

## Introduction

### Background and rationale {6a}

In a dynamic environment, the ability to rapidly process new information and flexibly adapt our behaviour and decisions to meet changing situational demands is crucial to survival. Cognitive flexibility (CF), one of the core components of executive functions (EFs) along with working memory and inhibition [1, 2], plays a pivotal role in enabling this adaptive capacity. CF is broadly defined as the mental ability to switch or shift between conceptual representations either spontaneously or in response to changing circumstances. CF has significant implications throughout the entire developmental lifespan and has been associated with multiple favourable life outcomes. In children and adolescents, higher CF has been linked to greater academic success, including improved reading, literacy and mathematics skills [3–6], better problem-solving abilities [7, 8] and a greater disposition toward critical thinking [9]. Notably, some of these associations between CF and academic outcomes have been found to persist into adulthood [10, 11]. Furthermore, higher CF is also associated with improved socioemotional skills and well-being such as better emotional regulation, higher emotional intelligence, and lower perceived stress in adults [12–15]. Finally, higher CF have also been found to mitigate age-related cognitive decline, contributing to better cognitive functioning and an improved quality of life among older adults [16–18]. Overall, these findings suggest the importance of CF for life success and overall well-being across the lifespan, highlighting its crucial role in our survival in a rapidly changing world.

The notion that cognitive abilities can be improved through training is rooted in the concept of brain plasticity, which holds that cortical representations are amenable and capable of neural reorganisation in response to novel learned experiences throughout any phases of life (for reviews [19, 20]). Recent decades have popularised the protective benefits of computerised cognitive training programmes in enhancing cognitive functions and combatting age-related cognitive decline. Despite an exponentially growing commercial demand, there is a lack of credible scientific evidence about their efficacy, and current findings have been mixed. Moreover, these programmes largely focused on working memory and inhibition as primary targets for cognitive training (for reviews [21, 22]) but showed minimal or short-lived near and far-transfer benefits to other cognitive skills aside from the trained cognitive function [23, 24].

Growing evidence suggests that training cognitive flexibility may be more effective, as compared to training working memory and inhibition. Using confirmatory factor analyses, Friedman and Miyake [25] demonstrated greater environmental relevance of flexibility-specific shifting factors relative to other EF factors. Despite limited studies investigating CF training, initial research suggests that CF training can lead to significant improvements in switching tasks and transfer benefits to other EF components [26, 27]. However, current CF training paradigms generally utilise traditional CF tasks, such as task set switching or the Dimensional Change Card Sort (DCCS) task [27–29]. CF tasks are inherently complex to tap on cognitive flexibility and this complexity also inadvertently recruit other EF components. Therefore, any observed training effect could be the compound effect of all three EFs, rather than solely CF. The current study thus plans to utilise a novel and more fundamental approach to improve CF with Structure Learning.

Structure Learning involves seeking patterns in the stochastic presentation of stimuli, without the need for explicit feedback. Under dynamic environments, Structure Learning encourages unbounded self-generation of rules or higher-order representations. Flexibility is an emergent consequence of exploring or updating rules generated in these environments. This approach hones a domain-general ability to abstract higher-order, ‘learning-to-learn’ principles, in response to a dynamically changing and uncertain environment, as opposed to promoting rote memorisation. The Structure Learning approach also stems from the idea that tasks can be organised in accordance to shared component processes. The experience of a variety of tasks with shared component processes can foster generalisable learning or more specifically, ‘meta’ learning (i.e. Structure Learning) [30, 31]. There is already emerging evidence that domain-general training of Structure Learning skills—for example through playing action video games—produces learning that transfers well beyond the training task [31]. Specifically, playing action video games that expose the player to multiple scenarios that all share a common structure has been associated with enhancements in cognitive flexibility and attentional control [32]. However, no existing studies have tested whether training in Structure Learning per se (as distinct from general video game playing) produces generalisable improvements in CF.

### Objectives {7}

The primary aim of the present study is to investigate whether a novel cognitive training programme based on Structure Learning can improve cognitive flexibility performance in a healthy adult population. The near-transfer benefits of the training are first evaluated within the Structure Learning task itself. We will investigate how participants adapt their behaviour in response to new stimuli and learning rules introduced intermittently throughout the training programme. We hypothesise that participants who undergo Structure Learning training will demonstrate the ability to apply previously extracted rules from their training sessions to these novel training elements. Secondly, near-transfer benefits are assessed within the CF construct using common tasks that measure CF performance. We anticipate significant improvements in CF performance among participants who underwent the cognitive training programme (Training group), in comparison to those who did not go through the programme (Control group).

The secondary aim of the study is to comprehensively assess potential behavioural and neural changes that may emerge from the cognitive training programme on a pilot level. We aim to investigate far-transfer benefits of the training across a wide range of socio-cognitive behaviours which includes working memory, inhibition, creativity, problem-solving, decision making and socioemotional skills. The Structure Learning task fundamentally taps onto the mechanism of statistical learning which underlies any learning or cognitive ability that involves probabilistic build-up of experiences or expectations. Hence, based on the ubiquity of the ability that the Structure Learning training programme aims to train, we hypothesise that we will see far-transfer benefits in at least one of the socio-cognitive domains that we are examining.

On a neural level, we employ a multimodality approach to examine potential neural benefits brought about by the cognitive training with both functional, i.e. resting-state functional magnetic resonance imaging (rs-fMRI) and magnetic resonance spectroscopy (MRS), and structural brain scans, i.e. multi-parameter mapping (MPM). Studies adopting training protocol based on Structure Learning have showed promising preliminary results of the potential neural benefits and demonstrated that the functional connectivity of the frontal-striatal circuits could predict decision performance [33, 34]. Building on findings from previous research, we hypothesise that we will see significant correlations of performance in the Structure Learning training programme with the frontal-cortico-striatal functional connectivity. Functional connectivity measures derived from rs-fMRI allow us to investigate functional networks within the brain but not discriminate between inhibitory and excitatory mechanisms. However, cognitive flexibility changes have been associated with neurometabolite changes in the prefrontal networks controlled by glutamatergic (excitatory) pyramidal neurons and GABAergic (inhibitory) interneurons [35–37]. Furthermore, GABAergic inhibition was found to optimise perceptual learning and decision making [38, 39]. Hence, the inclusion of MRS measurement of the neurotransmitter, γ-aminobutyric acid (GABA), will complement our findings in rs-fMRI and we hypothesise that higher GABA measured in the prefrontal cortex, an indication of better suppression of irrelevant information, will correlate with better Structure Learning performance. Previously thought to be a structurally permanent feature, recent evidence suggests that the rate and pattern of myelinisation in white matter can be modulated by learning and adaptation to novel experiences (for a review [40]). These changes, otherwise known as myelin plasticity, can play a pivotal role during learning, as well as after learning for consolidation. The present study will use MPM-R1 and R2* to probe myelinisation distribution as a measure of the speed of information processing [41] in response to environmental changes. Here, we hypothesise that MPM measures that reflect the extent of myelinisation in frontal-striatal regions, particularly within the cortex, will be associated with one’s performance during the Structure Learning training.

### Trial design {8}

Participants are recruited in batches consisting of up to 18 individuals. To establish a baseline, each batch of recruited participants undergoes a cognitive session that assesses their verbal and non-verbal intelligence, and baseline cognitive flexibility performance. Following the baseline assessment, participants are assigned to either the (1) Training group (receiving Structure Learning training) or (2) Control group (serving as a passive control without any training) using a parallel assignment method. The allocation ratio is set at 1:1 to ensure an equal distribution of participants between the two groups. Group assignment follows procedures of a randomised controlled trial but with matching constraints of age, sex, intelligence, and baseline cognitive flexibility performance between groups. Measures of executive functions can be susceptible to practice effects which can confound the study outcomes [42]. We circumvent this issue by administering most primary and secondary measures only at the post-test to both Training and Control groups. Therefore, matching the groups in the aforementioned variables is important so that we can compare critical outcome measures of the two groups at post-test to examine the effects of the Structure Learning training programme. Consequently, the study is designed as a superiority trial with the aim of demonstrating that the performance of the Training group that engages in the Structure Learning training programme will be superior to that of the Control group for primary outcome measures administered at post-test.

## Methods: participants, interventions and outcomes

### Study setting {9}

All cognitive-behavioural sessions are conducted in either the Yunnan Campus or Novena Campus of the Nanyang Technological University (NTU) in Singapore. The choice of the campuses depends on participants’ preferences and availability. Cognitive-behavioural sessions held at the NTU Yunnan campus would be carried out at the Centre for Lifelong Learning and Individualized Cognition (CLIC) whereas sessions held at the NTU Novena Campus would be carried out at the Lifespan Research Centre (LRC). All neuroimaging sessions are conducted at the Cognitive Neuroimaging Centre (CoNiC) that house a Siemens 3 T MAGNETOM Prisma MRI scanner. CoNiC is situated at the Experimental Medicine Building in the NTU Yunnan Campus.

### Eligibility criteria {10}

Inclusion criteria for the study are healthy volunteers of all sexes, aged between 18 and 55 years (inclusive), who have provided written informed consent to participate in the study. Exclusion criteria are as follows: (1) current and/or prior history of learning disabilities, neurological disorder, psychiatric disorder, and/or cardiovascular disorder; (2) predominantly left-handed; (3) contraindications for MRI safety (e.g. presence of pacemakers, implanted pumps, and/or metal objects in the body); (4) claustrophobia; (5) pregnancy; (6) lactation; and/or (7) pronounced visual or auditory impairments.

### Who will take informed consent? {26a}

Written consents are obtained from participants through two informed consent forms (ICFs) at two different time points. Firstly, a ‘cognitive ICF’ covering all cognitive-behavioural testing and Structure Learning training sessions is signed prior to the first Baseline Cognitive Session. Secondly, an ‘MRI ICF’ covering both the pre- and post-test neuroimaging sessions is signed at the start of the pre-test neuroimaging session.

During the informed consent briefing sessions, trained experimenters introduce participants to the study details and procedures, including the study’s group assignment, participant’s timeline, and payment details. Participants have the opportunity to read through the study information sheet and seek clarifications on any aspects. Written informed consent is obtained from participants once their understanding of the study process is confirmed after all concerns are addressed. In compliance with the Human Biomedical Research Act regulated by the Ministry of Health Singapore, a witness will be present during all briefing sessions to confirm voluntary participation and that participants are not coerced into participating in the study.

The informed consent briefing sessions for both the cognitive ICF and MRI ICF follow a similar structure, with differences in the methods of administration. The cognitive ICF session is conducted remotely using Zoom videoconferencing software, and consent is obtained using Adobe Acrobat e-signature software. The electronic cognitive ICF is sent to the individual participant, experimenter, and witness for sequential signing. The MRI ICF is conducted in-person during the pre-test neuroimaging session. During the MRI consent briefing session, participants receive additional information regarding the risks, potential of incidental findings, and safety considerations related to the MRI scans.

For participants below the age of 21 years, the informed consent process is additionally administered to one of their parents or guardians. A separate remote-guided informed consent briefing session is scheduled before the Baseline Cognitive Session. This session allows parents or guardians to provide consent for their child/ward and to clarify any questions they may have pertaining to experimental procedures that their child/ward will go through.

### Additional consent provisions for collection and use of participant data and biological specimens {26b}

During the informed consent process for both the cognitive ICF and MRI ICF, participants can indicate their preferences on the storage of their research data or health information for future research, and their willingness to be contacted for future research opportunities. Additional information is collected only when participants consent to any of these options, such as the specific types of research which they consent to their data being used for, and their preferred contact methods for future research.

## Interventions

### Explanation for the choice of comparators {6b}

A passive control was chosen as our only comparator without an active control in consideration of the following: (1) Our training programme is unprecedented, and we needed more data to further optimise it; hence, we prioritise the assessment of potential transfer effects and against a passive control without additional complexity from an active control. (2) We plan to examine potential neural changes resulting from the Structure Learning training. Given the novelty of Structure Learning, it is difficult to derive a definite brain network that is activated during Structure Learning with limited past studies to refer to. However, the choice of the active control task is critically dependent on knowledge of this network so that our imaging results are not confounded by the task used for the active control. (3) The inclusion of one more comparator would require additional trained research personnel to run the study, and this is logistically challenging given our intensive study schedule and limited manpower. Moreover, the present study is also conceptualised to approach that of a pilot study. Hence, we opted to have only one comparator. Nevertheless, it is noteworthy that we do have plans to include an active control condition in subsequent studies.

### Intervention description {11a}

**Fig. 1:**
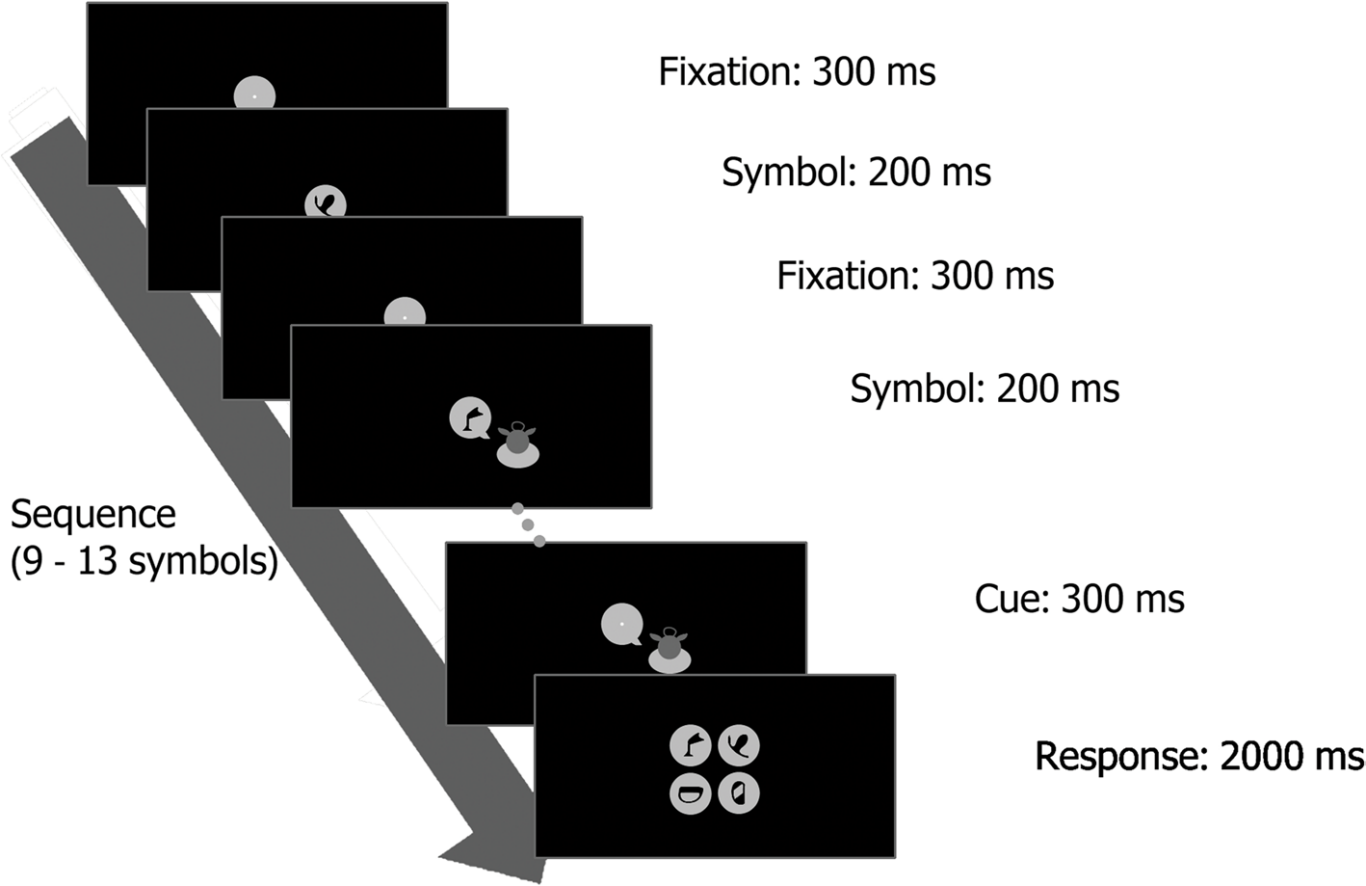
Trial sequence for the Structure Learning task

**Fig. 2:**
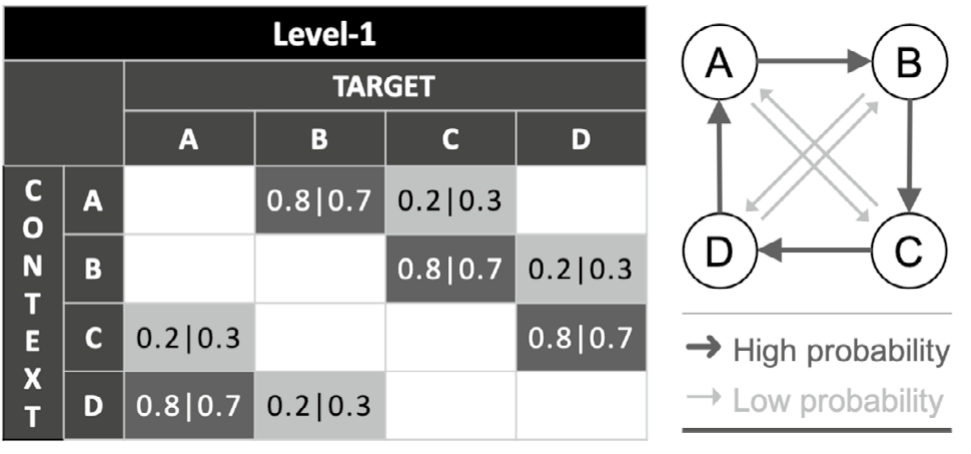
Sequence design for the Structure Learning task

Participants assigned to the Training group undergo a minimum of 13 Structure Learning training sessions. These sessions are conducted daily and guided remotely by trained experimenters. Each participant has an account pre-created for them on the iABC platform, hosted on servers under the University of Cambridge. This platform is the main interface for all Structure Learning training sessions and several post-test cognitive tasks. Each Structure Learning training session comprises 5 blocks of 60 trials each lasting around 10 min. Feedback on training performance is provided by the block. On each trial, participants are presented with a sequence comprising 9 to 13 symbols, lasting 200 ms each and asked to predict the next symbol at the end of the sequence within a response time window of 2 s (see Fig. 1 for detailed trial sequence). Symbols presented are from the obscure Ndjuka syllabary so that participants do not have prior exposure to them. The sequence of symbol presentation is determined by a Markov first-level model [34, 43] where the target depends on the symbol that immediately precedes it (see Fig. 2 for detailed contingency mapping). Each context is mapped onto two targets, one with a high presentation probability and one with a low presentation probability. The definition of high and low presentation probability, and hence difficulty level of each session, is manipulated and will vary across different training stages so that participants are challenged as they progress through the training sessions.

The intervention phase consists of four stages of Structure Learning, as illustrated in Table 1. This includes two training and two testing stages. ‘Training 1’ and ‘Training 2’ stages differ in terms of the stimuli used and the contingency ratio, with the former using a contingency of 80/20 and the latter using a contingency of 70/30. All participants start at ‘Training 1’ stage, and they progress to the subsequent stages (‘Testing 1’ > ‘Training 2’ > ‘Testing 2’) only if they achieve mean performance index (PI) of 75% on the last two blocks for two consecutive sessions in ‘Training 1’ stage. At each new stage, participants complete a mandatory practice session before proceeding with the actual training session.

**Table 1:** Structure learning training

Stage	Stimuli set	Sequence level	Conditions	Progression criterion	Session number
Training 1		1	Level 1 (80/20)	At least 75% for mean PI in the last two blocks across two consecutive sessions	6 ≤ x ≤ 12
Testing 1		1	Level 1 (80/20)	NA	1
Training 2		1	Level 1 (70/30)	NA	12 – x
**Total number of training sessions**					**12**
**After post-test MRI session**					
Testing 2		1	Level 1 (30/70)	NA	1

Table showing the stimuli sets and conditions administered for each stage of Structure Learning training. Each participant will complete at least stage 1 training and testing. If the progression criterion of stage 1 training is met, participant will then move on to stage 2 training and testing.

### Criteria for discontinuing or modifying allocated interventions {11b}

The current study recruits only healthy participants. Therefore, the intervention will only be discontinued if the participant chooses to withdraw from the study or if there is an unexpected scheduling conflict that prevents them from committing to the remaining experiment sessions.

### Strategies to improve adherence to interventions {11c}

The Structure Learning training programme is rigorous as it involves 1-h daily training for at least 13 consecutive days and can impact participants’ adherence to it. The study team has implemented the following steps to improve adherence and minimise participant’s withdrawal from the study. Firstly, the training is guided remotely, and participants do not need to attend training sessions physically. This enables greater flexibility in the scheduling of training sessions and ensures that training occurs in a comfortable and familiar setting for the participants. The remote-guided protocol used for the Structure Learning training programme is based on Leong et al. [44] who demonstrated comparable data quality between laboratory-based and remote-guided studies. Secondly, we also provided a comprehensive study timeline to participants at the first point of contact during the informed consent session to ensure participant’s availability for all sessions. Advance scheduling is done for all sessions to ensure that participants can plan their schedule accordingly in consideration of the sessions. Thirdly, we also ease the scheduling of sessions by assigning the same experimenter to each participant throughout the training phase. Lastly, continuous monitoring of participants’ attendance and training progress via a master schedule documenting all sessions and the experimenters-in-charge also enable prompt identification of any non-adherence and facilitate timely communication and support between experimenters. Besides appropriate arrangements to ease scheduling, we also monitor participant’s arousal, valence, and sense of control via the Self-Assessment Manikin [45] at the start of each training session. We can then quantify possible psychological effects from the training sessions and rectify any potential issues.

### Relevant concomitant care permitted or prohibited during the trial {11d}

The current study targets healthy adults unlike clinical trials that involve clinical populations. Hence, there are no alternative interventions for participants. Nonetheless, to ensure that any observed effects can be unequivocally attributed to the Structure Learning training programme, participants are asked to avoid engaging in any other intervention studies or brain stimulation research during their study involvement. We will proceed to withdraw a participant once we know that they are concurrently involved in other intervention studies or brain stimulation research to maintain the integrity of the data.

### Provisions for post-trial care {30}

We anticipate minimal harm to participants as the current study primarily involves cognitive training and tasks. However, we acknowledge that mental fatigue may arise with multiple experiment sessions that can span up to 3 h. Furthermore, our MRI scans are also longer than usual scans due to the sequence run. Thus, we enforce compulsory breaks during all experiment sessions whenever possible and participants are also allowed to take additional breaks during the sessions. Participants can also choose to withdraw from the study at any point in time. Even though MRI is in general a safe technique, the exposure of participants to a strong magnetic field can lead to minor side effects such as tingling sensations in the limbs or vertigo. Experimenters will closely monitor and follow-up on participants’ well-being after each MRI session. Serious adverse events will be reported promptly to the university’s IRB for appropriate action. Researchers may also unintentionally discover information about participants’ health conditions from their brain scans. Any brain structural anomalies observed will be raised to the principal investigators as incidental findings and reviewed by medical professionals. Barring any life-threatening medical issues, we only notify participants of any incidental findings if they indicate that they would like to be notified. The principal investigator(s) and/or qualified medical personnel(s) will explain the findings to the participants.

### Outcomes {12}

In the present study, multiple behavioural tasks are administered to measure various cognitive constructs. Primary behavioural constructs under investigation include Structure Learning, cognitive flexibility, inhibition, and working memory. Secondary behavioural constructs under investigation include creativity, intelligence, problem-solving, language skills, numeracy skills, social tendencies, and decision making. These measures are conducted only at pre-test assessment, i.e. baseline, only at post-test assessment, i.e. after the end of structure learning training and approximately 2 months from pre-test assessment, or at both pre-test and post-test assessments. Specifically, we administered secondary behavioural constructs measuring language skills, numeracy skills, and intelligence (with the exception of the Raven’s Advanced Progressive Matrices) only at pre-test assessments as they are traits that remain relatively stable across the adult lifespan. We administered primary and secondary behavioural constructs measuring cognitive flexibility (with the exception of the Colour Shape Task), inhibition, working memory, creativity, and problem-solving only at post-test assessments to eliminate possibility of practice effects. Finally, matching tasks, i.e. Colour Shape Task and Raven’s Advanced Progressive Matrices, secondary behavioural constructs that measure social tendencies and decision making (and hence more impervious to practice effects) and all secondary neuroimaging constructs are measured at both pre and post-test assessments. Both the Control and Training groups undergo the same measures at pre and post-test assessments. However, measures for Structure Learning are collected only for the Training group. In addition, we investigate potential neural benefits of the Structure Learning training by assessing secondary neuroimaging outcomes both structurally and functionally. Detailed information regarding the individual tasks used this study can be found in Appendix B in the supplementary information.

#### Primary outcomes

- (1) Cognitive flexibility
  Measuring cognitive flexibility poses a significant challenge due to the multitude of ways in which it has been defined and measured in previous research. In the present study, cognitive flexibility is operationalised by incorporating both set shifting and task switching behavioural paradigms. These paradigms have been widely utilised in numerous studies to investigate cognitive flexibility (for reviews [46, 47]). Although both set shifting and task switching taps onto cognitive flexibility, they invoke slightly different cognitive processes. In set shifting, participants are typically presented with the same stimuli sets throughout the task, which consist of two or more features. To successfully complete the task, participants must dynamically shift their attention between these features, generating different rules that enable them to effectively follow the overall instructions and solve the task. To investigate set shifting ability, the Wisconsin card sorting test (WCST), intra/extra-dimensional set shifting, and probabilistic reversal learning tasks are used. In task switching, participants are similarly presented with the same stimuli throughout the task but instead, explicitly asked to switch between tasks with different instructions. We used the task set switching where, task set switching what, colour shape task (CST), and trail making test to investigate task switching ability. Both conventional measures and modelling parameters will be derived from these tasks for further analyses.
- (2) Working memory
  The working memory component in the executive function framework derived by Friedman and Miyake[25] pertains more to the processing control and updating capabilities within working memory instead of its temporary storage capabilities. Hence, tasks chosen to investigate this updating aspect commonly necessitate additional processing of the encoded information. We chose three tasks to investigate working memory updating in the present study. In backwards digit span (BDS), participants are presented with an auditory stream of digits then asked to repeat them backwards. In the reading span task, a dual task paradigm is used, and participants have to alternate digits and sentence judgement which also invokes processing and updating capabilities of the working memory. In the Cambridge neuropsychological test automated battery (CANTAB) version of the spatial working memory task, participants are asked to continuously update spatial information encoded in their working memory as the task progresses to solve the task.
- (3) Inhibition
  Inhibition in the context of the executive function framework refers specifically to the ability to exercise control over one’s behaviour through overriding a prepotent and automatic response. Inhibition or response inhibition is particularly pivotal in the regulation of goal-directed behaviours. In the present study, inhibition is measured with the Stroop task and the stop-signal task.
- (4) Structure learning
  Structure learning is the training component of this study assessing through multiple measures. Contingency learning in participants is quantified as a performance index (PI) that reflects the comparison of participant’s response distribution with presented target distribution across the training. In addition, the types of strategy used by participants are also quantified with strategy choice and the strategy integral curve difference (strategy ICD). These variables are further used to examine the rate of change of learning and strategy across training sessions. Detailed calculations of these measures are included in the statistical plan.

#### Secondary behavioural outcomes

Secondary behavioural outcomes in the context of the present study are mainly outcomes that we are planning to investigate potential cross-domain far-transfer benefits of the Structure Learning training. These different tasks and key variables of interest are listed in Table 2.

**Table 2:** Secondary behavioural tasks and variables of study

Construct/domain	Task	Variables/subtasks
Demographics, Social & Psychological variables	Social questionnaires	• Perceived Stress Scale
		• Pittsburgh Sleep Quality Index
		• Empathy Quotient
		• Social Value Orientation
		• Prisoner’s Dilemma
		• Trust Game
		• Risk Preference
		• Ambiguity Aversion
		• Personal Relative Deprivation Scale
		• Cooperativeness and Competitiveness Personality Scale
		• Tolerance of Uncertainty
		• Multilingualism
		• Perceived Social Support
		• Big Five Inventory
		• Creative Mindset
Language & Numeracy	Woodcock Johnson IV (WJIV)	• Letter-Word Identification
		• Applied Problems
		• Passage Comprehension
		• Calculation
		• Sentence Reading Fluency
		• Maths Facts Fluency
General Intelligence	Raven’s Advanced Progressive Matrices (RAPM)	• Total correct
		• Change in accuracy score
	Wechsler Abbreviated Scale of Intelligence (WASI) Vocabulary	• Total score
	Wechsler Abbreviated Scale of Intelligence (WASI) Block Design	• Total score
Creativity	Verbal Fluency	• Mean fluency score
		• Clustering coefficient
		• Average shortest path length
		• Modularity
	Alternate Uses Task	• Total fluency score
		• Total originality score
		• Total flexibility score
	Remote Associates Test	• Percentage of correct solutions

#### Secondary neuroimaging outcomes

All structural and functional scans are acquired on a 3 T Siemens MAGNETOM Prisma MRI scanner (Siemens Medical Systems, Erlangen, Germany) with a 64-channel head coil. MRI scans were conducted at two timepoints, namely pre-test and post-test for all participants. The same scan sequence is used for all pre-test and post-test MRI scans within each participant. However, there can be changes in scan sequence between participants due to technical issues.

- (1) Resting-state functional MRI data
  rs-fMRI can reveal functional connectivity within and across brain networks that subserve task performance. A 10-min run of rs-fMRI data is acquired at both pre-test and post-test to investigate functional brain network changes that may result from the Structure Learning training. Before the rs-fMRI scan, participants are instructed to relax, fixate at a white cross presented at the centre of the screen, and to let their mind wander and not think about anything in particular. rs-fMRI images are acquired using Gradient Echo type Echo Planar Imaging (GRE-EPI) sequence with the following parameters: repetition time (TR) = 1280 ms; echo time (TE) = 30 ms; flip angle = 74^◦^; resolution matrix = 220 × 110 × 64; field of view (FOV) = 220 mm; thickness = 2.0 mm; acquisition voxel size = 2 × 2 × 2 mm^3^ resolution; integrated Parallel Acquisition Techniques = 4. A total of 64 slices are used to cover the whole brain including the cerebellum. Each 10-min run results in 462 volumes. In addition, high-resolution T1-weighted structure images are acquired using a Magnetisation Prepared Rapid Acquisition Gradient echo (MPRAGE) sequence with the following parameters: TR = 2000 ms; TE = 2.26 ms; inversion time = 800 ms; flip angle = 8°; FOV = 256 × 256; slices = 176; thickness = 1.0 mm; voxel size = 1 × 1 × 1 mm^3^).
- (2) Multi-parameter mapping
  MPM allows us to investigate different aspects of microstructural changes in grey matter and white matter. Recent evidence suggests that myelination is associated with behavioural plasticity in learning [41]. Hence, we focus on MPM parameters sensitive to myelin content, such as R1 and MT. All MPM scans are acquired with a 64-channel radio-frequency (RF) receive head coil and RF body coil for transmission. We use a multi-centre validated MPM protocol [48] in the present study. Three different multi-echo FLASH scans will be acquired with predominant T1-, PD-, and MT-weighting with appropriate choice of the TR and the flip angle. Multiple gradient echoes are acquired with alternating readout polarity at eight equidistant TE between 2.46 and 19.68 ms for the T1w, MTw, and PDw acquisitions. Acquisition parameters for T1-weighting and PD-weighting are as follows: voxel size = 1 × 1 × 1 mm^3^; slices = 176; FOV = 256; resolution matrix = 256 × 256; parallel imaging using GRAPPA factor in the phase-encoding (PE) direction = 2; 6/8 partial Fourier in partition direction, non-selective RF excitation, readout bandwidth BW = 480 Hz/pixel, RF spoiling phase increment = 50°. MT-weighting is achieved by applying an off-resonance Gaussian-shaped RF pulse (4 ms duration, 220° nominal flip angle, 2 k Hz frequency off set from water resonance) prior to the excitation. A pair of RF sensitivity maps are collected before each FLASH scans with the following parameters: 4 mm isotropic resolution, matrix = 256 × 64 × 48, parallel imaging using GRAPPA factor 2 in phase-encoding direction. The total acquisition time is around 15 min. The data-driven approach, i.e. unified segmentation-based correction of R1 maps for RF transmit inhomogeneities (UNICORT), is used to estimate B1 transmit bias field map [49].
- (3) Magnetic resonance spectroscopy
  MRS allows for non-invasive in vivo quantification of brain neurometabolites. In the present experiment, we targeted the measurement of the inhibitory neurotransmitter, GABA, in bilateral dorsolateral prefrontal cortex (DLPFC). Prior to all MRS scans, a high-resolution T1-weighted structural scan along the sagittal plane (MPRAGE; TR = 2000 ms; TE = 22.6 ms; TI = 800 ms; flip angle = 8°; FOV = 256 × 256; slices = 176; voxel size = 1 × 1 × 1 mm^3^) is acquired. We then 3D-reconstruct the axial and coronal planes and use images from all three planes to guide our voxel positioning on the DLPFC. We try to achieve consistent MRS voxel placement between subjects and sessions with the use of salient anatomical landmarks such as the dorsal anterior cingulate cortex, superior frontal gyrus, and middle frontal gyrus. In addition, we also try to maximise the sampling of grey matter in each region during voxel placement. After voxel placement, we ensure a homogeneous B_0_magnetic field within the DLPFC voxel with a two-step process. An automated B0 map-based shimming is first used to correct for shifts in linear gradients in the field. Subsequently, an interactive manual shimming procedure is used to correct for residual high-order heterogeneities in the B0 magnetic field. We aim to obtain an eventual shim that is as close to 16 Hz or preferably less than 16 Hz (full-width at half-maximum; FWHM) spectral linewidth. We then measured GABA levels with the widely used technique known as Mescher-Garwood Point Resolved Spectroscopy (MEGA-PRESS) [50, 51]. MEGA-PRESS implements J-difference editing to derive an edited signal that we then use it to quantify GABA levels. Two single voxel edited MR spectra are acquired from a 30 × 15 × 30 mm^3^ (in the *x*, *y*, and *z* dimensions) voxel of interest positioned separately in the left and right DLPFC. Two interleaved datasets are acquired within a single acquisition namely: (1) an edited ‘On’ inversion pulse at 1.98 ppm and (2) an ‘Off’ inversion pulse elsewhere at 7.5 ppm. Additional MEGA-PRESS parameters used are as follows: TR = 2000 ms; TE = 68 ms; spectral bandwidth = 1850 Hz; data points = 2048; readout duration = 1107 ms. We also acquire unsuppressed water spectra as a separate scan to allow for concentration reference to the water tissue. In total, 128 spectral averages (128 ‘On’ and 128 ‘Off’ pulses) are acquired during the metabolite scan and 4 spectral averages are acquired during the water scan resulting in a total scan duration of around 9 min.

### Participant timeline {13}

**Fig. 3:**
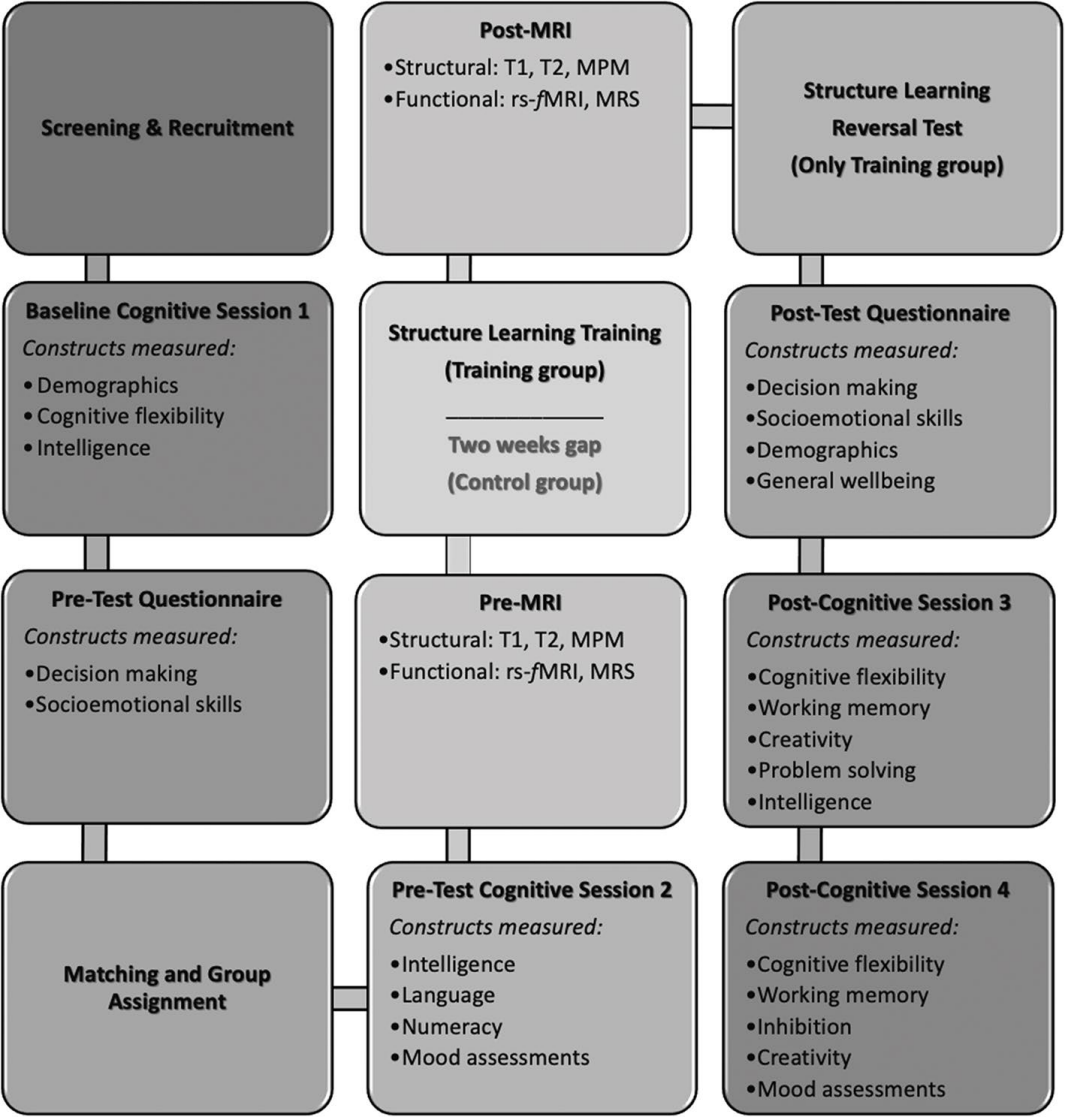
General participant timeline and sessions’ flow

Participants are recruited from various social media platforms. In addition, we also contacted higher education institutes such as polytechnics and universities in Singapore to send out email recruitment advertisements. Individuals who express interest in participating will be asked to complete an Eligibility Screening Questionnaire. Eligible participants who meet the predefined inclusion and exclusion criteria are contacted through email and invited to take part in the study. Participants follow the same schedule shown in Table 3, with slight differences depending on their group assignment. The study commences with the baseline assessments, comprising Baseline Cognitive Session 1 and Pre-test Social Questionnaires. Once the baseline cognitive measures have been administered, participants are pseudo-randomised into either the Training or Control groups. Participants then proceed to complete the remaining sessions outlined in Fig. 3. During the cognitive sessions, participants complete a comprehensive battery of cognitive tasks and questionnaires. In the pre-test, we focus on measuring various traits such as language skills, numeracy skills, and non-verbal intelligence. In the post-test, we administer critical outcome measures such as cognitive flexibility, working memory, inhibition, and other secondary behavioural measures. MRI sessions during both pre-test and post-test employ the same MRI scan protocol. During the 2-week interval between pre- and post-intervention phases, participants in the Training group additionally undergo a minimum of 13 Structure Learning training sessions. A detailed list of tasks that each participant will undergo at each session of the study can be found in appendix B from the supplementary information. The study is projected to span a total of 10 weeks. A time commitment of 12 and 32 h is needed for participants in the Control and Training groups respectively.

**Table 3:** Participant schedule

		**Study period**									
**Stage**	**Screening**	**Pre-test**					**Intervention**		**Post-test**		
		**Week**									
**Session**		**1**	**2**	**3**	**4**	**5**	**6**	**7**	**8**	**9**	**10**
Screening and Enrolment	B										
Baseline Cognitive Session 1		B									
Pre-Test Social Questionnaire		B									
Matching and Scheduling			B	B							
Pre-Test Cognitive Session 2					B	B					
Pre-MRI					B	B					
Structure Learning							T	T			
Post-MRI									B	B	
Structure Learning (Testing Stage 2)*									T		
Post-Test Social Questionnaire									B	B	
Post-Test Cognitive Session 3										B	B
Post-Test Cognitive Session 4										B	B

*B* Involve BOTH Control and Training Groups

*T* Involve Training Group ONLY

^*^ Only for Training participants who advanced to Training 2 stage

### Sample size {14}

The present study is designed as a scoping study to provide a good estimate of the sample size needed to detect the actual training effect in subsequent studies. Hence, sample size estimation may not be that accurate since the training programme implemented in the current study is novel and effect sizes can be hard to estimate.

Behaviourally, the sample size estimation is based on a statistical power of 0.8 with type I error rate held at an alpha level of 0.05. The estimates of statistical power are all made based on a balanced sample (equal sample size for all conditions) with a 2 × 2 mixed design with one between-subject factors (Group: Control and Training) and one within-subject factor (Time: Pre-test and Post-test). The detection of a Group × Time interaction with a small effect size of *d* = 0.25 and an assumed low population correlation of 0.3 between levels of the within-factor, Time, would need 46 participants for each level in the between-factor, Group.

Sample size estimation is done separately for the different neural measures. In healthy adults, GABA spectral peak height relative to that of water in the PFC generally showed a standard deviation of 0.24. Based on past MRS studies that measure GABA in DLPFC, the detection of a small effect size in GABA spectral peak height relative to water would need 50 participants for each group. In rs-fMRI, based on a reported correlation of *r*= 0.46 between the functional connectivity of corticostriatal networks and performance measures for Structure Learning [33], we derived a sample size of 35 participants per group to detect significant correlations. Finally, as MPM is still a relatively novel scan sequence used to quantify microstructures within the brain, prior data is hence unavailable for us to perform any power computation and sample size estimation. Instead, we sampled published MPM studies that employed similar methodological analyses [52, 53] and opted for an average sample size of 34 participants per group.

Based on sample size computation of the various measures, we opted for the highest sample size of 50 participants per group to ensure sufficient statistical power to detect significant effects in all measures. With a data attrition rate of 20%, the required sample size is set at 60 participants per group.

### Recruitment {15}

A multi-pronged approach is used for recruitment. This includes placement of physical posters across different university campuses and disseminating recruitment advertisements on social media platforms such as Telegram and Facebook. Participants are screened twice to ensure that they meet the inclusion and exclusion criteria of the study. In the first screening round, participants are requested to complete a brief online questionnaire. This questionnaire collects basic screening criteria such as age, sex, nationality, information on pre-existing health condition, and critical MRI contraindications. Experimenters will carefully review responses from the first screening round and invite eligible participants to proceed to the second round of screening,

In the second round of screening, participants are invited to complete a more detailed online screening questionnaire. This questionnaire includes more in-depth health screening questions and the Edinburgh Handedness Inventory [54] to gather additional information on participants’ eligibility. After reviewing responses, eligible participants are recruited in batches and promptly contacted to schedule the first Baseline Cognitive Session. This batch recruitment approach ensures effective management and allocation of available manpower and facilitates the matching of participants into Training and Control groups. Participants who do not meet the eligibility criteria will be informed.

## Assignment of interventions: allocation

### Sequence generation {16a}

The allocation sequence is controlled with an algorithm written in R and we employ variance minimisation procedures [55] to match age, sex, intelligence and cognitive flexibility while randomly allocating participants to either the Training or Control group. Intelligence is assessed using the RAPM score, while cognitive flexibility is assessed through the switch cost in accuracy and reaction time on the CST. Both intelligence and cognitive flexibility measures are collected during the Baseline Cognitive Session. The matching algorithm script is executed only when participants within a recruitment batch have all completed the Baseline Cognitive Session. Data from the previous batches of recruited participants is considered during the group allocation. This approach ensures that the two groups remain matched on pre-specified matching variables with each batch of incoming new participants.

Participant data for each matching measure are first collated into a source Excel file. Group assignment for the first recruitment batch is done sequentially upon initialisation of the algorithm. Once the number of assigned participants reaches the number of groups present, i.e. two in the present study, subsequent participants are temporarily assigned to one group at a time. For each potential group assignment, we compute the mean of each matching measure within each group and thereafter derive a mean of means between groups as well as the sum of squared deviations of this group mean. The end goal of the algorithm is to achieve the best minimal sum of squared deviations of this group mean by exploring all possible group allocation of a particular participant. We ensure a balance group allocation of 1:1 (Training vs. Control) by constraining the algorithm such that it will not assign a new participant to the same group as the previous participant.

### Concealment mechanism {16b}

Since the allocation sequence is generated using an algorithm, the group assignments are unpredictable and unknown to participants and experimenters until after the script is run. This takes place at least 2 weeks after the Baseline Cognitive Session. The group assignment is done once all participants of the same batch have completed the Baseline Cognitive Session. Prior to that, participants are assigned an enrolment ID that runs chronologically in the sequence of their recruitment. Hence, both experimenters and participants are unaware of participant’s group assignment until the allocation is completed.

### Implementation {16c}

A dedicated smaller team of research personnel is responsible for conducting the Baseline Cognitive Session and running of the group allocation script. This restricts data access to confidential participant data to a selected few within the research team. In addition, as the Baseline Cognitive Session involves mostly standardised tasks, all responsible research personnel are intensively trained in both administration and scoring of the tasks to ensure consistency. Before the allocation process, participants will be assigned a recruitment ID indicating their recruitment batch. Once participants have been assigned to a specific group, a new participant ID that differentiates participant by their group assignment will be provided. Participants will be notified of their group assignment via email or phone and asked to complete an online survey to facilitate scheduling of the remaining sessions.

## Assignment of interventions: Blinding

### Who will be blinded {17a}

The study adopts a single-blind design with participants blinded to the full details of the Structure Learning training programme under investigation. Participants are informed that they are either in Group A or B without any knowledge of whether that refers to the Training or Control group. Training participants, i.e. participants in Group A, will complete the appropriate Structure Learning stage during each training session without knowledge of the stage progression criterion and the context-target stimuli contingency. Participants are only told to predict the next symbol as accurately as possible based on previously appearing ones. This is especially crucial for the study as it investigates whether cognitive flexibility can be improved via implicit learning from Structure Learning training. Control participants, i.e. participants in Group B, are unaware of the Structure Learning training programme. During the pre- and post-cognitive sessions, the construct measured by each task is non-explicit and never mentioned in the instructions given to the participants (e.g. ‘WCST is a card-sorting task’). This is to prevent task expectations from affecting participants’ performance. The group information is not blinded for experimenters during the administration of tests and data collections. However, manuals to standardise experimenters’ interactions with and instructions to the participants are prepared and intensive training provided for all experimenters to ensure consistency in all administered procedures. Researchers performing the data analysis will not be blinded to the group assignments. However, researchers’ biases are prevented during data analyses by setting hypotheses in advance with sufficient sample size planned prior to data collection. All final analysis scripts will be shared on open science platforms or repositories. Due to ethical regulations, the full data set will not be made available publicly. However, regulated data access can be provided to other researchers under request.

### Procedure for unblinding if needed {17b}

Upon completion of the study, participants will be debriefed via email. This debriefing will inform them about their group assignment, i.e. Training or Control in the study. Additionally, participants will be informed of the purpose of the Structure Learning training programme as well as the cognitive tasks that were conducted during the study.

## Data collection and management

### Plans for assessment and collection of outcomes {18a}

Prior to data collection, all experimenters received training to provide standardised instructions and to follow the standard operating protocol (SOP) when administering the tasks. The training sessions spanned 12 h across 4 to 5 days to cover a brief background of the study, SOPs for all tasks in cognitive sessions and Structure Learning training session, as well as safety precautions and preparation work for MRI sessions. During the training, experimenters also had the chance to try out each task and practice giving instructions to their partners (other experimenters undergoing training). Manuals containing SOP for each task were available to experimenters in Teams online and printed out to be used during cognitive sessions. The manual contains the exact instructions that experimenters have to read out, with points to be emphasised bolded in text, as well as other set ups to be standardised (e.g. volume of instructions to be played for CANTAB tasks, baseline and ceiling scoring). This allows experimenters to familiarise themselves with task administration, and to give consistent instructions to all participants. After the training session, newly recruited experimenters will be observed by a full-time research staff during their first cognitive, training, and MRI sessions with participants to ensure that the protocol is executed correctly and to provide any additional feedback. Most cognitive tasks are computerised to improve protocol adherence, with the exception of WASI Vocabulary, WASI Block Design, WJIV tasks, verbal fluency task, and BDS. This also reduces the need for manual data entry so that there would be less errors.

Standardised tests used to measure intelligence (RAPM, WASI Vocabulary, WASI Block Design), language, and numeracy skills (WJIV tasks) are widely validated. The RAPM administered in pre-test and post-test are parallel versions that contain items from Set I and Set II of RAPM [56]. The pre-test and post-test versions have Cronbach’s *a*= 0.59 and 0.64 respectively [57]. No concurrent validity was reported during the construction of Zaaiman et al.’s [57] version of RAPM, but items in both versions were selected using empirical data. Only the vocabulary and block design subtests of WASI-II are administered to measure verbal and non-verbal intelligence respectively. Both subtests have excellent internal reliabilities and high concurrent validity, with Cronbach’s *a*ranging from 0.90 to 0.92 and correlation with other intelligence tests ranging from 0.71 to 0.92 [58]. Lastly, the WJIV tasks also exhibit reliability coefficients ranging from 0.74 to 0.97 and have strong concurrent validity with other tests [59].

Preprocessing will first be performed on rs-fMRI data to enhance quality of data. A standard SPM pipeline (http://www.fil.ion.ucl.ac.uk/spm/software/spm12/, SPM12 v7771) will be employed to model fMRI data. We will reslice the aligned EPI data to 2 × 2 × 4 mm^3^ resolution and apply spatial smoothing with a 6-mm isotropic FWHM Gaussian kernel (SPM smooth). The rs-fMRI noises will be processed by standard denoising pipeline in CONN (https://web.conn-toolbox.org, CONN 21a), including linear detrending, 12 potential motion related noise components, noise components from cerebral white matter and cerebrospinal areas, wavelet despiking. Note that the global signal will not be added as a nuisance regressor as suggested by Murphy and Fox [60]. We also correct for inhomogeneity of the magnetic field by acquiring opposite phase encoded field maps (phase encoded direction A-P and P-A). FSL (version 6.0, using topup function) will be used to preprocess the field maps.

Similarly, processing steps will be conducted concurrently with data analysis of MPM to promote data quality in MATLAB 2020b using hMRI (https://hmri-group.github.io/hMRI-toolbox/). In brief, regression of the log signal from the echoes of all weighted volumes will be used to calculate a map of R_2_* using the ordinary least squares ESTATICS approach [61]. Then the set of 8 echoes for each acquired weighting will be averaged to increase the signal-to-noise ratio. Quantitative R1 maps will first be estimated based on the Ernst Eq [62]. and then further corrected for transmit field inhomogeneities and imperfect RF spoiling [63]. The effective transverse relaxation rate (R_2_^∗^) will be estimated from the logarithm of the signal intensities (from the 8 PDw multi-echo images) at different echo times using a linear regression. Effective PD maps will be estimated from the signal amplitude maps by adjusting for global and local receive sensitivity differences using UNICORT post-processing approach [49]. As the global mean PD cannot be estimated accurately with this post-processing approach, we will scale PD value to 69% of mean white matter. Finally, the MT map will be constructed using the procedure described by Helms, Dathe, and Dechent [64]. After the MPM map are created, segmentation will be performed (grey matter, white matter, and cerebrospinal fluid) combining information from MT and R1 maps. Next, we will perform normalisation by aligning all images to the MNI space in preparation for group analyses. DARTEL (SPM12) will also be implemented to improve alignment across subjects / sessions when normalising to MNI space. Finally, we will apply tissue-specific smoothing on the segmented four MPM maps using voxel-based quantification (VBQ) smoothing with 6-mm FWHM. Smoothing is performed to improve the alignment across participants and boost the signal-to-noise ratio (SNR) for group analyses. MPM values extracted are all based on the smoothed and segmented MPM map.

The MRS data will also be preprocessed using Osprey [65] with MATLAB 2020b. The 128 edit-on and 128 edit-off data will be corrected for eddy-current correction, frequency-and-phase correction, and Fourier transformed prior to grouping on and off spectra and taking the corresponding edit-on and -off spectra differences.

### Plans to promote participant retention and complete follow-up {18b}

During the consent-taking process, participants are informed about the maximum time commitment needed for the study. This ensures that participants can commit to all the sessions in the study regardless of their group assignment and to reduce participant withdrawals after the groups are assigned. Furthermore, immediately upon group assignment, participants will be scheduled for all subsequent sessions. This minimises the chances of session cancellations since participants can do advance planning of their own schedule. Experimenters will send reminder emails and messages before each session to ensure that participants are aware of and well-prepared for the upcoming session. Moreover, they will also actively engage with the participants throughout the study and provide flexible alternatives for rescheduling whenever possible.

### Data management {19}

Researchers from the NTU-CLIC team will be responsible for data entry and analyses. Participants will be assigned a subject identification number so that data collected at different stages of the study can be linked to the corresponding participant. Records of potential participants that had contacted the recruitment team to express their interest in joining the study will be kept in an Excel sheet to ensure that progress and status of recruitment are being tracked closely. This Excel sheet includes the number of potential participants that contacted the recruitment team, inclusion/exclusion criteria status, withdrawal, and completion of the study participation.

Data collected as part of the research participation will be downloaded from the respective platforms used. Non-computerised tasks will be manually recorded and keyed into Excel spreadsheets. Quality checks will be performed routinely by the research team to ensure that data is proper. The data are then kept on a secured server that only the research team and other approved personnel can access. The researchers are responsible for the data maintenance and analyses.

### Confidentiality {27}

The data collected has been kept securely in a server that is accessible only by the IRB-approved research team and approved personnel. A list of personnel who are allowed access is submitted to the NTU-IRB. Additionally, the data collected are in accordance with Singapore’s Personal Data Protection Act. Participants will be assigned an identification number that are with no relation to identifiable personal data. A list of ID matching to each participant’s personal data are kept separate and are only accessible by a few personnel in the research team.

### Plans for collection, laboratory evaluation and storage of biological specimens for genetic or molecular analysis in this trial/future use {33}

N/a. Biological specimens are not collected in this study.

## Statistical methods

### Statistical methods for primary and secondary outcomes {20a}

#### Quantification of Structure Learning training outcomes

Most Structure Learning training outcomes are computed as in Wang et al.. [34, 43] As the Structure Learning training programme assesses contingency learning, trial task accuracy will not be useful to assess learning. Instead, learning is assessed in a probabilistic manner. We will compute a PI for each context within each training block (see Fig. 2 for more information on the context-to-target contingencies) to quantify the minimum overlap between the participant response distribution and the presented stimuli target distribution. The overall PI for a specific training block is then derived by taking the contingency-weighted average PI across all possible contexts. We will also adjust this PI measure for random guess baseline to derive a normalised PI measure.

Besides assessing learning, we will also quantify the main strategies employed by the participants during Structure Learning training sessions. The Kullback–Leibler (KL) divergence index will be used to compare participant response distribution to a probability matching and probability maximising distribution separately. Participants who engaged in greater matching will have a response distribution that closely matches the presented target distribution across all context-target pairings, i.e. if context A is followed by target B 80% of the time and target C 20% of the time, participant will pick target B in 80% of the trials and target C 20% of the trials for a perfect matching response distribution. Participants who engaged in greater maximising will have a response distribution where the most likely outcome is always chosen, i.e. if context A is followed by target B 80% of the time and target C 20% of the time, participants will always choose target B for a perfect maximising response distribution. The model difference quantified by KL divergence index is referred to as strategy choice wherein negative values indicate a strategy closer to matching and positive values indicate a strategy closer to maximising. A strategy choice is derived per training block and strategy fluctuations can be tracked across training blocks and sessions for each participant. We will then attempt to derive a general index that can reflect participant’s selection of strategy for each training session. For each individual participant, we will compute this strategy index, herein termed strategy integral curve difference (strategy ICD) by taking the difference between the integral of each participant’s strategy curve with the integral of the exact matching curve.

#### Evaluation of efficacy of Structure Learning training

We will primarily evaluate the efficacy of Structure Learning training by examining differences in post-test measures for EF between Control and Training groups. Measures will be either standardised for easier interpretations or normalised whenever possible. Means and standard deviations of either a suitable population reported in published studies or the large-scale study that is concurrently carried out by CLIC to characterise the latent construct of CF, herein termed as WP0.1, will be used for data normalisation. Parametric tests will be used to compare group differences of primary outcome measures whenever possible. Besides focusing on individual primary outcomes, we will also examine group differences with a combination of primary outcome measures that best characterise the latent constructs of cognitive flexibility, working memory, and inhibition in the structural equation modelling outcomes of WP0.1. A multivariate mixed effects model will be used to account for the nested data structure and to investigate differences between Training and Control group on these combined EF measures.

#### Examining performances in Structure Learning training and its relation to cognitive flexibility

We will examine how performances in Structure Learning training affect post-test cognitive flexibility measures. As participants go through multiple training sessions, they will need to learn varying underlying contingencies and adjust their strategies in order to solve the task. A mixed effects model will be used to model the changes in PI and strategy ICD across training sessions so that we can examine how these changes affect post-test CF measures. Measures collected during the Baseline Cognitive Session 1 and Pre-test Cognitive Session 2 will be entered as covariates in the model.

#### Examining functional connectivity network changes from the Structure Learning training

Functional brain networks will be extracted through spatial group independent component analysis (GICA) in CONN [66]. Preprocessed EPI data from Training and Control groups in both sessions, i.e. Pre-test and Post-test, will be included in the GICA. We will first use principal component analysis to reduce dimensionality at both participant and group levels. Participant-specific spatial maps for each component will then be subjected to GICA3 back reconstruction to estimate spatial independent neural patterns for identification of functional brain networks [66]. We will then compute intrinsic and extrinsic connectivity to examine changes in functional connectivity, i.e. post-test minus pre-test and its relation to the Structure Learning training measures. Intrinsic connectivity is computed based on the correlation between filtered time course of each voxel with every other voxel in the participant-specific component. Extrinsic connectivity is computed based on the correlations of time series between cortical ICA components with striatal regions defined using the Melbourne Subcortex Atlas (https://www.nitrc.org/projects/msa) [67]. All correlation matrixes will be standardised with Fisher *z*-transformation and averaged to derive a mean index of functional network connectivity for each participant and session. These are then correlated with behavioural index of Structure Learning performance, such as strategy index with the Robust correlation toolbox [68].

#### *Examining bilateral dorsolateral prefrontal (DLPFC) GABA* + *concentration changes from the Structure Learning training*

The basis set of brain model metabolite spectra from LCModel (version 6.3) will be used to fit preprocessed MRS data to derive GABA + concentration from the right and left DLPFC. We will model creatine(tCre), choline(tCho), glutamate, Glx(Glu + glutamine), and NAA from the edit-off spectrum, and GABA + from the difference spectrum. Resultant model fits for the MRS data will be assessed for quality based on Cramer-Rao Lower Bound (CRLB) values of less than 20% for bilateral DLFPC and sessions and signal-to-noise ratios of > 20. GABA + concentration differs between tissue types. We will employ a tissue correction strategy to correct for the dependency of GABA + on voxel tissue composition. The percentage of grey matter (GM), white matter (WM), and cerebrospinal fluid (CSF) in each of the measured voxel during MRS will be computed and GABA + concentration will be divided by GM/(GM + WM + CSF). Similar to the planned analysis for rs-fMRI, we will then correlate the changes in corrected GABA + concentration, i.e. post-test minus pre-test with behavioural index from the Structure Learning training with the Robust correlation toolbox [68].

#### Examining changes in myelinisation distributions in frontal-striatal regions from the Structure Learning training

Parameter maps will be generated based on Weiskopf et al [48]. After normalisation and smoothing, we will extract mean MPM measures from masks of ICA components derived from the rs-fMRI GICA analysis. Difference scores from MPM parameter maps will be correlated with behavioural indexes computed from Structure Learning training to investigate the relationship between myelinisation distributions with how one performed during the Structure Learning training. We are expecting significant correlations to manifest in cortical regions. 

### Interim analyses {21b}

Two interim analyses have been conducted separately for behavioural and neuroimaging data before the end of data collection. For the behavioural interim analyses, we checked through all tasks on the different platforms used for data collection and visually inspected them to ensure that there were no technical errors such as network issues that resulted in data loss. The behavioural data is mainly accessed by CL, XC, JYJT, WLK, and MBU. For the MRI interim analyses, basic preprocessing and data quality checks are done to detect possible technical errors or excessive movement artefacts during scans so that we can sift out unusable participant data. As such data will have to be discarded, this will affect whether we achieved adequate sample size to detect the effects that we would like to investigate. The MRI data is mainly accessed by CL, MH, JYJT, BLC, and XC. Data collection will be terminated formally once we reach sufficient usable participant data as stipulated in our sample size estimation for all primary and secondary outcome measures.

### Methods for additional analyses (e.g. subgroup analyses) {20b}

#### Examining strategy-associated changes during Structure Learning training in cognitive flexibility and functional connectivity

In addition to the planned analyses mentioned previously, we will also explore data-driven approaches with rs-fMRI data. These exploratory analyses (connectome-based predictive modelling) can complement results from the planned analyses and ascertain whether findings from both approaches corroborate. The Structure Learning training protocol that we are implementing is a novel cognitive training approach. Thus, our hypotheses are formulated based on limited past studies and it is hard for us to make predictions on the type of decision strategies that participants will engage in and how this will affect our behavioural and neuroimaging outcomes. Published studies on Structure Learning generally suggest the usage of two main strategies as an individual goes through Structure Learning training. Hence, we will also conduct subgroup analyses to examine the impact of different decision strategies on cognitive flexibility primary outcomes and functional connectivity patterns derived from rs-fMRI.

#### Subgroup categorisation of decision strategies

We will categorise participants of the Training group into three groups, i.e. maximising, matching, random based on their mean strategy ICD across the training sessions which indicates the strategy that they primarily engaged in. Criterion for the strategy group categorisation will be based on similar past studies [33, 34, 43, 69]. However, due to sample size constraints, minor modifications may be made to ensure that each strategy group has adequate participants to perform group comparisons. We will then examine how these different strategy groups can affect functional connectivity pattern changes and cognitive flexibility outcomes.

### Methods in analysis to handle protocol non-adherence and any statistical methods to handle missing data {20c}

Any deviations from the protocol due to human or technical errors are noted down in an Excel sheet and corrected whenever possible. Missing data in behavioural outcomes are accommodated with the use of mixed effects models. Data imputation will be performed only if we have more than 20% of data missing for any one of the behavioural measures. We will not perform any data imputation for neuroimaging data. Instead, any unusable data in a particular MRI modality will be discarded and neuroimaging data from other intact MRI modalities kept for analyses. Hence, the eventual number of participants used for analyses in the different neuroimaging modalities may differ. Finally, all data from any participants who have withdrawn from the study will be excluded from the data analyses.

### Plans to give access to the full protocol, participant-level data, and statistical code {31c}

The full protocol including MRI processing pipelines and statistical code will be shared on open science platform (https://osf.io/) and/or GitHub. Pre-prints of findings will first be uploaded to preprint servers, such as bioRxiv (https://www.biorxiv.org/) and then published to peer-review journals. All data (both behavioural and neuoimaging data) generated from this research are subjected to restrictions by the National Research Foundation (NRF), Singapore and Nanyang Technological University (NTU), Singapore, and hence not publicly available. Any data required to support the protocol, are, however, available from the authors upon reasonable request and with the permission of both NRF and NTU.

## Oversight and monitoring

### Composition of the coordinating centre and trial steering committee {5d}

The researchers from the NTU-CLIC are responsible for the study’s day-to-day data collection, data analysis, and organisational supports to ensure the smooth running of the study. The principal investigators have a monthly meeting with the researchers, during which they are updated on the study’s progress and to discuss data analysis plans.

### Composition of the data monitoring committee, its role and reporting structure {21a}

N/a. Since the current study implements cognitive training, there is no risk of causing clinical disturbance; hence, we decided to not have a data monitoring committee.

### Adverse event reporting and harms {22}

Adverse events are collected and assessed on its severity by the principal investigator and researchers of this study. Should there be an incident of a serious adverse events or an unanticipated problem during the data collection process, the principal investigator of the study will report it to the NTU-IRB by submitting the details of the event in an Incident Report Form. An unanticipated problem will also be submitted to the IRB if there is any unforeseen harm that may or may not occur that is inconsistent with the risk levels previously approved for the study. This includes issues of confidentiality and complications in using the study’s materials and devices.

### Frequency and plans for auditing trial conduct {23}

There was an IRB compliance auditing check requested by NTU-IRB on 22 July 2022. All research team members were required to attend and display all consent forms, every member’s research training certificates, and data management method for a check. Access to data and personal information was also checked to ensure the adherence of NTU-IRB policy.

### Plans for communicating important protocol amendments to relevant parties (e.g. trial participants, ethical committees) {25}

Protocol amendments to the study are only submitted to the NTU-IRB via the university’s ethical review management portal. The ethics committee from the university’s research integrity and ethics office review approve or request for additional changes to the submission via this portal as well. Once submitted, all investigators and key personnel of the study have been informed of this submission via email. After approval, the investigators and key personnel are notified via email and the study protocol on the university’s ethical review management portal is updated to include the amendment changes as an updated version. This study protocol as well as the email approval letter have been downloaded and stored in a cloud-based storage service for record keeping purposes.

### Dissemination plans {31a}

The study has been pre-registered on the National Institutes of Health U.S. National Library of Medicine ClinicalTrials.gov, identifier ID: NCT05611788. The research group also plans to present the study’s core findings to the stakeholders and the public through international peer-reviewed articles and conferences. Findings will also be shared with our participants upon their request with identifiable details being masked.

## Discussion

This paper outlines the protocol for conducting a Structure Learning training programme and the evaluation of its effectiveness with regard to various cognitive domains. Participants are matched based on age, sex, intelligence, and cognitive performances in a Baseline Cognitive Session and assigned equally into either a Control or Training group. Both groups will then be required to complete a pre-training cognitive assessment that comprises of standardised tests that measure intelligence, and language and numeracy skills. Following that, participants undergo the pre-training MRI scans. During a period of 2 weeks, the Training group commences on their Structure Learning training sessions, whereas the Control group will not be given any form of training. Post-training MRI scans will be taken immediately after the end of the 2-week gap. Finally, two post-training cognitive sessions will be conducted for both groups of participants.

As this study is conducted in a single-blind manner, participants are not privy to the group they are assigned to, while experimenters are aware of the group participants are in. This may result in an increased likelihood of experimenter bias, where the experimenter may unknowingly provide certain cues or lead participants in a certain way during Structure Learning training sessions or cognitive sessions. Additionally, there are certain cognitive assessment that requires a slightly level of subjective judgement in scoring participant responses; preconceived notion about the group assigned to the participant may cause experimenters to have slight differences when scoring these responses.

The Structure Learning training outlined in this study is designed to target improvements in CF, as well as potential transfers to other cognitive functions. As highlighted earlier in this paper, cognitive flexibility is an important cognitive function, contributing to better adaptability in dynamic changes in environment [70] and other important activities of daily living. The development of a training will be critical with higher attentions to improve cognitive capabilities across the ages. Additionally, the correlating of behavioural training outcomes to neurological model can also contribute to better understanding of neuropsychological model of learning. This paper will also serve as a basis of reference to assist other researchers in designing their study.

## Trial status

Study recruitment began in May 2022 and data collection formally started in June 2022. The research team experienced multiple technical difficulties in the implementation of the planned scan sequences between July 2022 and October 2022. Data collection was paused and study protocol revised to resolve these issues. First registration of the revised version of the study protocol on ClinicalTrials.gov was completed on 7th November 2022. The current version of the study protocol will be in use until the end of our data collection projected to be in June 2023.

### Supplementary Information

**Additional file 1. **Appendix

## Data Availability

Any data required to support the protocol are available from the authors upon reasonable request and with the permission of both NRF and NTU.

## Abbreviations

CF: Cognitive flexibility
CLIC: Centre for Lifelong Learning and Individualised Cognition
DLPFC: Dorsolateral prefrontal cortex
EF: Executive function
EPI: Echoplanar imaging
FOV: Field of view
FWHM: Full-width at half-maximum
GABA: γ-Aminobutyric acid
GICA: Group independent component analysis
Glx: Glu + glutamine
GM: Grey matter
ICD: Integral curve difference
IRB: Institutional review board
MPM: Multi-parameter mapping
MRS: Magnetic resonance spectroscopy
NTU: Nanyang Technological University
PI: Performance index
R_2_: Transverse relaxation rate
RF: Radio-frequency
rs-fMRI: Resting-state functional MRI
SNR: Signal-to-noise ratio
tCho: Choline
tCre: Creatine
TE: Echo time
TI: Inversion time
TR: Repetition time
VBQ: Voxel-based quantification
WM: White matter
