# Supplementary material for: Potential cognitive and neural benefits of a computerised cognitive training programme based on Structure Learning in healthy adults: study protocol for a randomised controlled trial
Source: Trials. 2023 Aug 11;24:517. doi: 10.1186/s13063-023-07551-2 (PMC10422731; doi:10.1186/s13063-023-07551-2)
Supplement: Supplementary file 1 — Additional file 1. Appendix [file 13063_2023_7551_MOESM1_ESM.pdf]

Appendix A: Tasks conducted at each study session

Baseline Cognitive Session 1

- Cognitive Flexibility
  - Colour Shape Task
- Intelligence
  - Raven’s Advanced Progressive Matrices
  - WASI Vocabulary

Pre-Test Social Questionnaires

- Decision Making
  - Social Value Orientation
  - Prisoner's Dilemma
  - Trust Game
  - Risk Preference (Positive)
  - Ambiguity Aversion
  - Socioemotional skills, beliefs and tendencies
  - Creative Mindset
  - Receptiveness to Opposing Views
  - Need for Closure Scale (Short)
  - Personal Relative Deprivation Scale
  - Cooperativeness and Competitiveness Personality Scale
  - Empathy Quotient (EQ)
  - Curiosity and Exploration
  - Racial Essentialism Scale

Pre-Cognitive Session 2

- Intelligence
  - WASI Block Design
- Language and Numeracy
  - Woodcock-Johnson IV
- Mood Assessments and Control
  - PHQ-9 (Patient Health Questionnaire-9)
  - GAD-7 (Generalized Anxiety Disorder-7)

Pre-MRI & Post-MRI Sessions

- Structural
  - T1
  - T2
  - MPM
- Functional
  - rs-*f*MRI
  - MRS

Post-Test Social Questionnaires

- Demographics
  - Demographic and SES (Part of CLIP-Q)
  - Residential Characteristics (Part of CLIP-Q)
  - Clip-Q Singapore Language History Questionnaire
  - Decision Making
  - Social Value Orientation
  - Prisoner's Dilemma
  - Trust Game
  - Risk Preference (Positive)
  - Ambiguity Aversion
  - Socioemotional skills, beliefs and tendencies
  - Creative Mindset
  - Receptiveness to Opposing Views
  - Need for Closure Scale (Short)
  - Personal Relative Deprivation Scale
  - Cooperativeness and Competitiveness Personality Scale
  - Empathy Quotient (EQ)
  - Curiosity and Exploration

- Racial Essentialism Scale   - Big Five Inventory (10-items)   - General well-being   - Perceived Stress Scale   - Pittsburgh Sleep Quality Index   - Multidimensional Scale of Perceived Social Support
Post-Cognitive Session 3
- Cognitive Flexibility   - Intra-Extra Dimensional Task (IED)   - Wisconsin Card Sort Task   - Task Set Switching “Where”   - Task Set Switching “What”   - Creativity   - Alternate Uses Task   - Verbal Fluency   - Working Memory   - Spatial Working Memory   - Backward Digit Span   - Problem Solving   - One Touch Stockings of Cambridge   - Intelligence   - Raven’s Advanced Progressive Matrices
Post-Cognitive Session 4
- Cognitive Flexibility   - Probabilistic Reversal   - Trails Making A/B   - Colour Shape Task   - Working Memory   - Reading Span   - Inhibition   - Stop Signal Time Task   - Stroop   - Creativity   - Remote Associates Test   - Mood Assessments and Control   - PHQ-9 (Patient Health Questionnaire-9)   - GAD-7 (Generalized Anxiety Disorder-7)

Appendix B: Tasks used for primary and secondary behavioural outcomes

Primary outcomes

Primary behavioural outcomes

1. Structure Learning

Structure learning is the intervention administered to Training participants. The performance index (PI) is an index to quantify the minimum overlap in distribution of participant responses and distribution of the presented targets per context. It is further transformed to give relative PI, which normalises PI by subtracting out PI by random guessing. Strategy choice is the difference between the Kullback-Leibler (KL) divergence from model matching to the response-based model, and the KL divergence from model maximization to the response-based model. The integral curve difference is the integral of each participant's strategy curve that is further subtracted from the integral of the exact matching curve. In addition, we also calculated learning rate and strategy shifting rate to better track the learning progress, by taking the difference of PI and of integral curve difference across sessions (e.g., final session – initial session).

2. Wisconsin Card Sorting Test (WCST)

The Wisconsin Card Sorting Test (WCST) is a neurocognitive task commonly used in research and clinical settings (Miles et al., 2021). The task is frequently used to assess cognitive flexibility. In this study, participants are required to sort a cue card into 4 different piles of card (1 red triangle, 2 green stars, 3 yellow plus-shapes, and 4 blue circles). The cue card can be presented in various numbers (1 to 4), form or shape (triangle, star, plus-shape, circle), and color (red, green, yellow, blue). Participants will have to select the correct pile of card based on a rule unknown to them, feedback will be given after each choice to indicate if it was correct or wrong.

We will be incorporating a sequential learning model to compare between the Training and Control groups on structure learning outcome. The learning rate and decision consistency of the individual will be extracted from the model. Learning rate indicates how quickly participants update beliefs about the values associated with choices following feedbacks given. Decision consistency represents the estimated probability of the individual choosing a specific stimulus for each trial; a larger value indicates that there is an increase in exploitation and lower values indicate increased in exploration. Additionally, the proportion of perseverative errors, representing the proportion of trials where participants chose an incorrect deck based on the rule from the preceding trial, will be tabulated for analyses.

3. Intra-extradimensional set-shifting (IED)

Intra-extradimensional set-shifting (IED) is another task to measure cognitive flexibility as it involves change in rules throughout stages of the task. The task begins from simple discrimination/reversal, to compound discrimination/reversal, then intra-dimensional shift/reversal and eventually extra-dimensional shift/reversal stages (Jazbec et al., 2007). Throughout the nine stages, there were either changes in contingency or target stimuli, hence cognitive flexibility is crucial for responding correctly to each trial. Of particular interest to the study is the extra-dimensional set errors which tracks number of incorrect responses made after an extra-dimensional shift occurs. Similarly, extra-dimensional reversal errors are also included in analysis as a metric for accuracy after a rule reversal following extra-dimensional shift. Using the feature reinforcement model, three additional parameters are extracted, namely the learning rate, choice determinism and dimension primacy. Learning rate is the rate of learning the rules. Choice determinism measures the dependence of choices on stimuli and context presented, so higher choice determinism suggests that participants tend to respond to more frequently correct stimuli, whereas lower choice determinism leads to more random responses.

Dimension primacy refers to the amount of attention placed on the previously relevant dimension and ignoring the newly introduced dimension. As IED task involve rule-learning via feedback, the results are also modelled using the reinforcement learning (RL) framework (Sutton & Barto, 1998; Wilson et al., 2014; Nussenbaum & Hartley, 2019), particularly focusing on learning rate and explore-exploit parameters.

4. Probabilistic Reversal Learning (PRL)

Probabilistic Reversal Learning (PRL) is similar to other cognitive flexibility task and consists of discrimination and reversal stages whereby a rule reversal occurs. Variables relating to number of trials and responses to a rule switch are analysed. Perseveration refers to the number of trials until participant updates their response after the rule reversal. Switch probability is defined as the number of switches in participants' responses after negative feedback. The number of trials before participants reach the learning criterion during reversal stage of PRL are also used for comparison between groups. PRL results are processed with Reinforcement Learning Model, and extracted parameters include learning rates, reinforcement sensitivity and stickiness. Two learning rate parameters are extracted, one during positive feedback condition, and the other during negative feedback condition. Reinforcement sensitivity is the extent that participants' responses are determined by the previous reinforcements received. Lastly, there are two stickiness parameters – stimulus stickiness, which measures the tendency to respond with the same stimulus as previous trial regardless of the outcome; and location stickiness, which is the tendency to choose the stimulus at the same location of the previous response.

5. Task Set Switching (TSS) – Where and What

Task-set switching (TSS) is a common paradigm considered to be a measure of cognitive flexibility (Schmitz and Kramer, 2023). In our study, participants are required to attend to a cue stimulus that will indicate whether to pay attention to the letter or number of the test stimulus. Participants are then required to identify if the attended stimulus is on the left or right for the where version of the task. The What version of the task requires participant to identify if the letter is a consonant or vowel, or if the number is odd or even. Performances in response times and error rates of individuals will be analysed to compare effects of structure learning (Schmitz and Voss, 2014). In our study, we will be calculating the switch cost of both responses time and error rates. Switch cost in our study refers to the differences between switch and repeat tasks.

6. Colour Shape Task (CST)

The Colour Shape Task (CST) is a neurocognitive task commonly used to study cognitive flexibility as it assesses the ability of an individual to switch between mental operations. In this task, participants are given a cue to either pay attention to the colour or shape of a stimuli. They are then required to provide a response based on the given cue and features of the stimuli. Shift trials are trials with a different cue from its previous trial whereas repeat trials have the same cue as its previous trial (Miyake et al., 2004; Friedman et al., 2008). The switch cost in reaction time and accuracy are calculated as the difference in reaction time between the shift and repeat trials and difference in accuracy between the two trials respectively.

7. Trail Making Test (TMT)

Trail Making Test (TMT) is a widely used neuropsychological assessment to measure set shifting and hence cognitive flexibility. There are two trails completed sequentially in this test - Trail A, which requires participants to connect circles containing numbers in ascending order (e.g., 1,2,3) and Trail B, which requires connecting circles containing numbers and alphabets in alternative and ascending

order (e.g., 1,A,2,B) (Reitan,1955; Reitan 1958; Tombaugh, 2004). The primary outcome for this task is the ratio of completion time of Trail B to Trail A, where a change in instruction occurs from Trail A to Trail B.

8. Backwards Digit Span (BDS)

Backwards digit span (BDS), which measures the ability to recall presented digits in reverse, is a well-established working memory task (Giofre et al., 2015). The total number of correctly recalled sequences will be used for analysis.

9. Reading Span

The reading span task is commonly used as a measure of working memory (Chein and Morrison, 2010). We will be using a complex reading span task instead that is a variation of the reading span task. Participants will be shown digits followed by a sentence to be judged if it makes sense or not. At the end of each trial, participants will have to recall the digits in the presented order. This variation measures both the processing ability and information storage capacity of the individual (Unsworth et al., 2009). The total number of correctly recalled digits across the entire task will be used to compare working memory performances between the Control and Training group.

10. Spatial Working Memory (SWM)

The spatial working memory (SWM) task is used to provide measure of working memory errors (Cacciamani et al., 20187). Participants are tasked to search for tokens that are hidden in boxes presented on a screen, additionally instructed not to return to boxes that already had tokens in them. Between search errors, which indicates the number of times the participant revisits a box where a token had already been found at, will be tabulated for analysis. Additionally, the strategy score, indicating the number of times the participant begins a new search within the same trial from the same box, will also be obtained. For both variables, a lower value indicates better performances.

11. Stroop

The Stroop test is a typically used for measuring interference control (Gajewski et al., 2020; Stroop, 1935). We will be using a computerised version for conducting the Stroop test. Participants are required to enter the correct input according to the color of the presented word whilst ignoring the meaning of the word. Congruent trials are words presented in the same color as its meaning, and incongruent trials are words presented in a differing color than its meaning. Response time and proportion of correct trials will be measured as part of the outcome variables. Difference between incongruent and congruent trials will be calculated for data analyses.

12. Stop Signal Task (SST)

The stop signal task (SST) is typically used to measure the response inhibition in individuals (Lipszyc and Schachar, 2010). The probability of go responses on stop trials is collected as an outcome variable. Additionally, an estimation of the covert stop signal reaction time (SSRT) using an integration method (Verbruggen et al, 2019) is extracted for analysis.

Secondary outcomes

13. Social questionnaires

A collection of questionnaires is administered to participants pre- and post-intervention to gather their demographic information, as well as other social and psychological variables that may be used as covariates in future statistical modelling. The secondary measures are mainly from the following questionnaires or tasks: Perceived Stress Scale, Pittsburgh Sleep Quality Index, Empathy Quotient, Social Value Orientation, Prisoner's Dilemma, Trust Game, Risk Preference, Ambiguity Aversion, Personal Relative Deprivation Scale, Cooperativeness and Competitiveness Personality Scale, Tolerance of Uncertainty, Multilingualism, Perceived Social Support, Big Five Inventory and Creative Mindset.

14. Woodcock Johnson IV (WJIV)

Woodcock-Johnson IV (WJIV) is a comprehensive set of assessments used to measure academic abilities. This study employsed a subset of the WJIV's tests of achievement (ACH) to measure reading and math abilities during pre-test cognitive session. These tests are Letter-Word Identification, Passage Comprehension, Sentence Reading Fluency, Applied Problems, Calculation and Math Facts Fluency test. The first three tests measure reading abilities and the last three tests measure math abilities (Schrank, McGrew & and Mather, 2014). There are two secondary outcomes, the standardised literacy and numeracy score, which are generated after combining the reading and math tests scores respectively.

15. Raven's Progressive Advanced Matrices (RAPM)

The Raven's Progressive Advanced Matrices (RAPM) is a standard test for general intelligence, and a variation of the test developed by Zaaiman and colleagues (2001) is used in this study to minimize practice effects between pre- and post-intervention administration of the test. The number of correct responses is collected as raw measure of the test performance, and a change in scores is calculated as the difference between post and pre scores.

16. Wechsler Abbreviated Scale of Intelligence (WASI) Block Design

The Wechsler Abbreviated Scale of Intelligence (WASI) Block Design also measures general intelligence, specifically the ability to analyse and synthesise abstract visual items. The total score is recorded and standardised to T-score by taking into account participants' age.

17. Wechsler Abbreviated Scale of Intelligence (WASI) Vocabulary

The Wechsler Abbreviated Scale of Intelligence (WASI) Vocabulary is a test for verbal intelligence. The total score is recorded and standardised to T-score by taking into account participants' age.

18. Verbal fluency

Verbal fluency tasks are widely used to measure verbal functioning (Shao et al., 2014) and creativity (Silvia et al., 2013). In this study, we will be using both semantic and phonemic verbal fluency tasks. "Animals" will be used for semantic verbal fluency, and the letters "F", "A", and "S" for phonemic verbal fluency. Mean fluency scores will be obtained for analyses purposes, as well as parameters extracted from a Semantic Network Model. Additionally, responses will be timestamped for an optimal foraging model.

19. Alternate Uses Task (AUT)

Alternate Uses Task (AUT) is a common creatively test used to measure divergent thinking or the ability to generate multiple ideas after being introduced to a particular stimulus. In this study, participants were will be given images of six different items, one at a time. They had will have two minutes per item to list as many creative ways they could use this item (e.g., brick as a dumbbell) (Guilford, 1967; Kleibeuker et al., 2016). This task was is also only administered during the post-test and the study looks at three secondary outcomes, namely the fluency score (number of responses), total originality score (number of unique responses in relative to other participants) and total flexibility score (number of different categories or domains of responses).

20. Remote Associates Test (RAT)

Remote Associates Test (RAT) is also a well-known test for creativity in experimental psychology and is used to measure participants' ability to link or associate between seemingly unrelated words (Mednick, 1962; Mednick, 1968, Lee, Huggins, & and Therriault, 2014). In this study, participants were will be given three words and asked to provide a fourth word that can combine with each of the three words to form a compound word or phrase. Each participant will be given 30 sets of these three words, one at a time, and will need to provide their forthfourth word within one minute. This task was is also only administered during the post-test and the study looks at one secondary outcome, the percentage of correct responses.
